# Calcium Chaos in Sarcoidosis: A Tale of Severe Hypercalcemia's Diagnostic Challenge

**DOI:** 10.7759/cureus.56271

**Published:** 2024-03-16

**Authors:** Mansi Satasia, Archit Garg, Kulani Weerasinghe, Chirag Patel, Mario Caldararo

**Affiliations:** 1 Internal Medicine, Saint Peter’s University Hospital, New Brunswick, USA; 2 Pulmonary and Critical Care Medicine, Saint Peter’s University Hospital/Rutgers Robert Wood Johnson Medical School, New Brunswick, USA

**Keywords:** intrathyroidal tracer uptake, 1.25- dihydroxy vitamin d, atypical presentation of sarcoidosis, sarcoidosis hypercalcemia, extrapulmonary manifestation of sarcoidosis, chronic granulomatous diseases, severe hypercalcemia, pulmonary sarcoidosis

## Abstract

Sarcoidosis is a systemic inflammatory condition characterized by noncaseating granulomas. Lung involvement is typical, while extrapulmonary manifestations, notably lymphadenopathy, are observed in a significant proportion of cases. The etiology involves complex interactions among immune cells and mediators, resulting in granuloma formation capable of independently producing 1,25-dihydroxyvitamin D, leading to unregulated hypercalcemia and hypercalciuria. Diagnosis can be challenging, especially when hypercalcemia is the initial symptom. The presence of noncaseating granulomas on biopsy is characteristic of sarcoidosis.

We present a case of severe hypercalcemia in a 53-year-old woman, initially suggestive of primary hyperparathyroidism due to non-suppressed intact parathyroid hormone (PTH) levels and unilateral intrathyroidal tracer uptake on a technetium 99m sestamibi parathyroid scan. The patient presented with hypertension, acute kidney injury, and severe hypercalcemia. Initial assessment, including a parathyroid scan, hinted at primary hyperparathyroidism. However, further evaluation, including chest computed tomography (CT) and endobronchial biopsy, revealed sarcoidosis with noncaseating granulomas. Prednisone therapy led to normalization of serum calcium and creatinine levels.

The case underscores the complexities in diagnosing sarcoidosis, especially when presenting with severe hypercalcemia. Despite non-suppressed PTH and suggestive imaging, the final diagnosis relied on endobronchial biopsy findings. The study highlights the limitations of conventional diagnostic markers, emphasizing the need for a comprehensive and individualized approach.

## Introduction

Sarcoidosis, a multisystem inflammatory disease, is characterized by noncaseating granulomas. In the United States, it is more common in African Americans, with a slight female predominance [1]. It can affect any organ, with the most common being the lungs which are involved in over 90% of cases, with about 30% presenting extrapulmonary manifestations, prominently featuring lymphadenopathy [1]. Elusive etiology involves a complex interplay of immune cells and mediators, leading to the formation of granulomas capable of producing 1,25-dihydroxyvitamin D independently of parathyroid hormone (PTH). The unregulated hypercalcemia and hypercalciuria in sarcoidosis result from granulomas lacking negative feedback mechanisms [2]. Diagnosing sarcoidosis becomes challenging when severe hypercalcemia is the presenting symptom. Diagnosis requires a thorough evaluation, including PTH, 25-hydroxyvitamin D, and PTHrP assessments and biopsy. 1,25-dihydroxyvitamin D testing is crucial due to varying levels of 25-hydroxyvitamin D [2].

## Case presentation

A 53-year-old woman, a non-smoker, presented to the emergency department after noticing a low pulse oximeter reading (in 80s) at home. The patient, born in Hungary, worked as a kindergarten teacher and immigrated to the USA in 2013. The patient reported a chronic cough since childhood, worsening over the past six months, associated with exertional dyspnea. The dry cough, occasionally producing scant mucus, exacerbated in the morning without specific triggering or relieving factors. Over the last three months, she experienced dyspnea with routine activities previously manageable without shortness of breath. She also reported fatigue over the last three months. She had no increased thirst or urination, abdominal pain, nausea, bone pain, muscle weakness, and confusion. Review of the system was negative for fever, chills, sick contacts, sore throat, wheezing, heartburn, asthma history, chest pain, palpitations, recent surgery, prolonged immobilization, long travel, hemoptysis, night sweats, appetite changes, vomiting, constipation, or significant weight fluctuations. Medication included over-the-counter vitamin C and D supplements.

**Figure 1:**
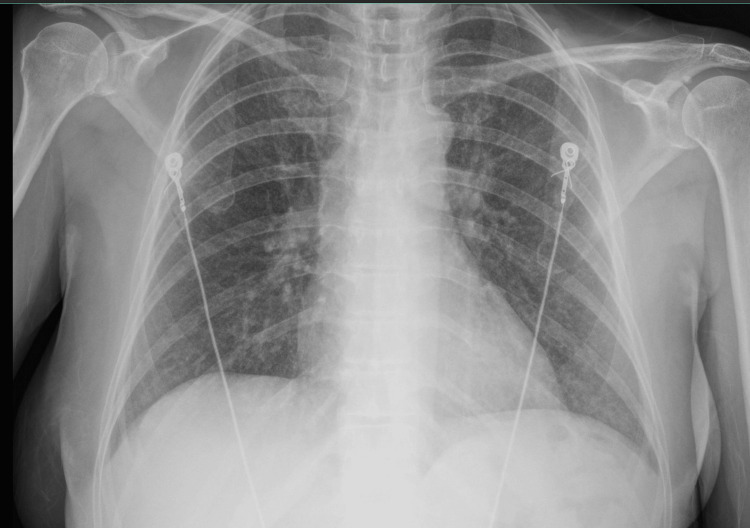
Chest X-ray showing bilateral pulmonary interstitial infiltrate

In the emergency department, her sensorium was intact, though affect was anxious. Her vitals including oxygen saturation were normal except for hypertension (190/97 mm Hg). Physical examination was otherwise unremarkable. Initial evaluation revealed elevated creatinine of 3.34 mg/dl (ref. range: 0.52 -1.04) and severe hypercalcemia of 18.1 (ref. range 8.4-10) along with normal albumin of 5.2 (ref. range: 3.5-5.0), ionized calcium 6.3 (ref. range: 4.5-5), blood urea nitrogen (BUN) 36 (ref. range: 6-20), creatinine of 3.34 mg/dL (ref. range: 0.52-1.04 mg/dL), estimated glomerular filtration rate (eGFR) of 14 (ref. range: >60), phosphorus of 4.2 mg/dL (ref. range: 2.7-4.5 mg/dL), bicarbonate of 35 mEq/L (ref. range: 21-33 mEq/L). Baseline laboratory values were not available prior to the initial presentation. Chest X-ray showed bilateral interstitial infiltrates as shown in Figure 1. She was admitted for severe hypercalcemia with hypertensive emergency.

Further extensive workup for secondary causes of hypercalcemia showed 25-hydroxyvitamin D of 64 ng/mL (ref. range: 25-80 ng/mL), an intact PTH (iPTH) of 15.7 pg/mL (ref. range: 7.5-53.2 pg/mL) with elevated total 1,25-dihydroxyvitamin D of 181 (ref. range: 18-72). 24-hour urinary calcium level (304) ruled out Familial Hypocalciuric Hypercalcemia. Renal Doppler and urinary and plasma metanephrines were normal so pheochromocytoma and MEN syndrome were not considered. PTH-related peptide of 18 (ref. range: 11-20) pointing against tumor-related hypercalcemia. Additional labs showed normal serum and urine protein electrophoresis, erythrocyte sedimentation rate (ESR) 33 (0-20), antinuclear antibody (ANA) negative, C3 121 (88-165 mg/dl), C4 54 (14-44 mg/dl), antineutrophil cytoplasmic antibody (ANCA) negative, and additional studies performed were negative for infection, autoimmune conditions, and malignancy. Thyroid functions and vitamin A were also within normal range. Serum immunofixation showed a normal pattern, and no monoclonal proteins were detected. ANCA was also negative. Laboratory values are shown in Table 1 below.

**Table 1:** Biochemical Study BUN: blood urea nitrogen; eGFR: estimated glomerular filtration rate; PTH: parathyroid hormone; ESR: erythrocyte sedimentation rate; ANA: antinuclear antibody; ACE: angiotensin-converting enzyme; TSH: thyroid-stimulating hormone

Laboratory Analysis	
Calcium	18.1 mg/dL (ref range: 8.4-10 mg/dL)
Albumin	5.2 g/dL (ref range: 3.5-5.0 g/dL)
Ionized Calcium	6.3 mg/dL (ref range: 4.5-5 mg/dL)
Phosphorus	4.2 mg/dL (ref range: 2.7-4.5 mg/dL)
BUN	36 mg/dL (ref range: 6-20 mg/dL)
Creatinine	3.34 mg/dL (ref range: 0.52-1.04 mg/dL)
eGFR	14 ml/min/1.73 m^2^ (ref range: >60 ml/min/1.73m^2^)
Bicarbonate	35 mEq/L (ref range: 21-33 mEq/L)
Intact PTH (iPTH)	15.7 pg/mL (ref range: 7.5-53.2 pg/mL)
25-Hydroxyvitamin D	64 ng/mL (ref range: 25-80 ng/mL)
1,25-Dihydroxyvitamin D	181 pg/mL (ref range: 18-72 pg/mL)
Exogenous 1,25-Dihydroxyvitamin D2	<8 pg/mL
Urine Calcium	13.8 mg/dL
24-hour urinary calcium level	304 mg/day (ref range: 35-250 mg/day)
PTH-related peptide	18 pg/mL (ref range: 11-20)
ESR	33 (0-20)
ANA	negative
C3	121 mg/dL (ref range: 88-165 mg/dL)
C4	54 mg/dL (ref range: 14-44 mg/dL)
Vit A	76 mcg/dL (ref range: 38-98 mcg/dL)
ACE	46 U/L (ref range: 9-67 U/L)
TSH	0.555 uIU/mL (ref range: 0.465-4.680 uIU/mL)
FT4	1.73 pg/mL (2.77-5.27 pg/mL)
FT3	3.11 ng/dL (ref range: 0.78-2.19 ng/dL)

**Figure 2:**
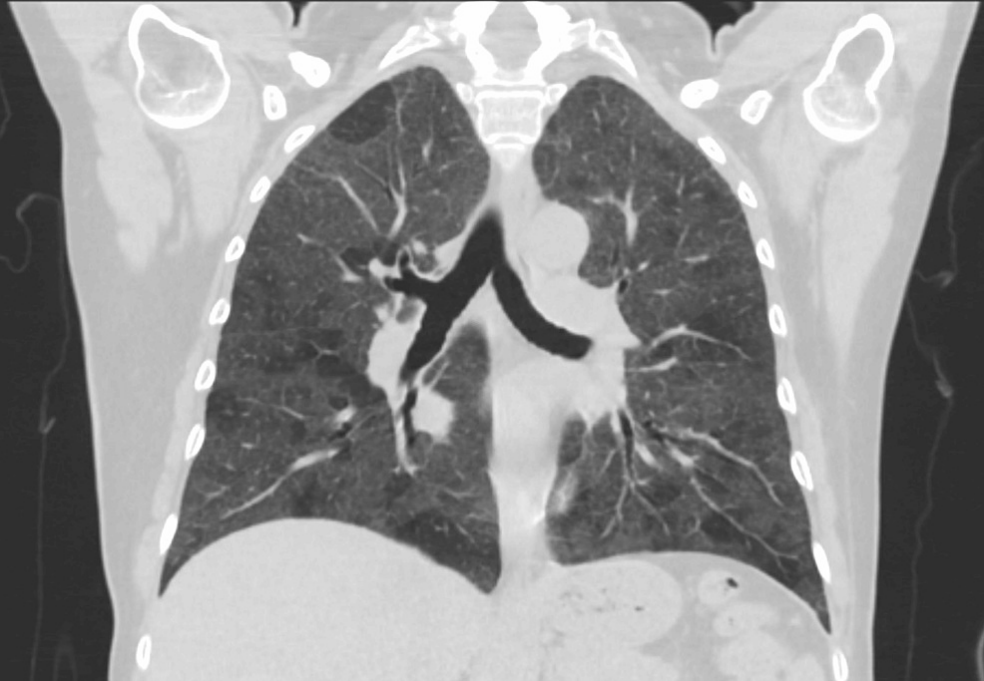
CT chest showing hazy ground glass opacity bilaterally

Based on initial labs and imaging, to evaluate for possible sarcoidosis, a CT thorax was done which showed a mosaic pattern with mild diffuse ground glass opacity in the lung fields as shown in Figure 2.

ACE level was 46 (ref range: 9-67 U/L) which was within normal limits. Aspergillus antigen and antibody profile were negative.

This case highlights a complex presentation involving respiratory symptoms, chronic cough, and severe hypercalcemia, prompting further investigation into the underlying etiology.

**Figure 3:**
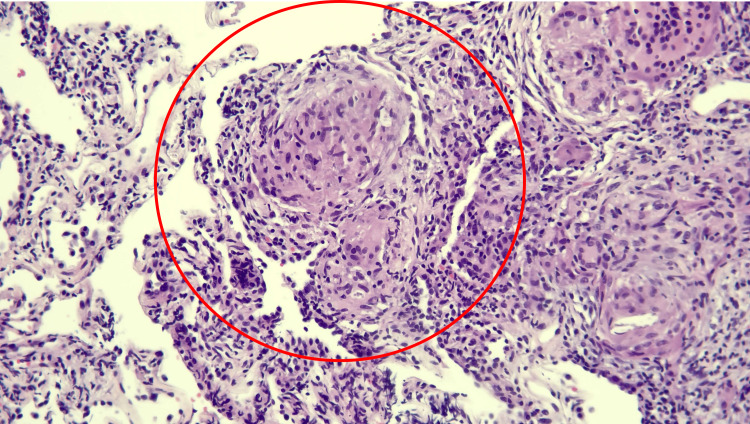
Biopsy from lung showing non-caseating granuloma

Bronchoscopy and the biopsy from RUL posterior lateral segment showed alveolar and endobronchial tissue with non-necrotizing granuloma and chronic inflammation as shown in Figure 3.

Flow cytometry of bronchoalveolar lavage showed a 62% T-cell count and CD4:CD8 ratio of 4.5:1, suggestive of sarcoidosis. Immunohistochemical stains (AE1/AE3, TTF-1, CD3, CD20, CD30) results support the diagnosis.

**Figure 4:**
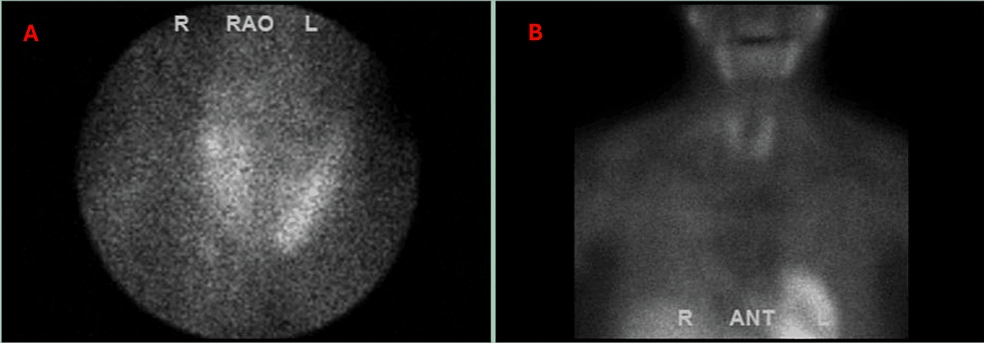
Tc-99 Sestamibi scan showing intrathyroidal tracer uptake in left inferior pole (A and B)

A technetium 99m sestamibi parathyroid scan showed unilateral intrathyroidal (left, inferior pole) tracer uptake concerning for possible parathyroid adenomas as shown in Figure 4.

**Figure 5:**
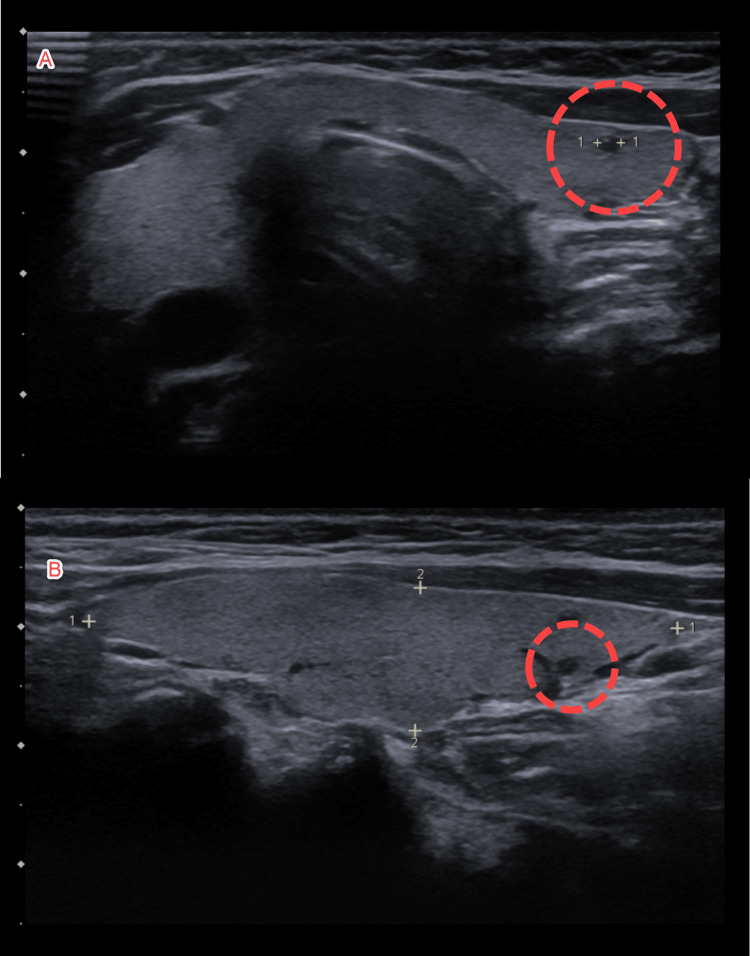
Thyroid ultrasound showing two nodules in the left upper pole (A and B)

For confirmation, a thyroid ultrasound was ordered which demonstrated two, 2x2x2 mm nodules in the left upper pole of the thyroid as shown in Figure 5, which did not correlate with the regions of uptake on the single photon emission computed tomography (SPECT), suggesting parathyroid adenoma as a cause of severe hypercalcemia least likely.

Initial treatment began with aggressive IV hydration, Zoledronic acid, and calcitonin. With a biopsy diagnostic for sarcoidosis, Glucocorticosteroids were started, initially intravenous (Solumedrol IV every 8 hours for six days) and then oral with a slow taper (Prednisone PO 30 mg daily for one week followed by 10 mg taper every one week until 10 mg dose, then 10 mg daily for two weeks tapered to 7.5 mg daily).

Three months after initial evaluation, the serum calcium level was 9.6 mg/dL, and creatinine was 1.1 mg/dL. The patient reported her shortness of breath resolved and her calcium level remained within normal range.

## Discussion

Sarcoidosis is a multi-system non-necrotizing granulomatous disease. In the US, African Americans have almost double the incidence of sarcoidosis as compared to Caucasians, followed by Hispanics and finally Asians [1]. The incidence varies regionally, with the highest rates in Northern European countries and the lowest in Eastern Asian countries [3]. It is accounting for 2-160/100,000 cases worldwide with a five-year mortality rate of approximately 7% [4]. The average age of diagnosis is 50, however, males often present between the ages of 20-45 while females present later at the ages of 50-65 [5]. The patient in this case report is a 50-year-old female from Hungary. Sarcoidosis is characterized by pulmonary or thoracic lymph node involvement in 90% of cases; eye, skin, and peripheral lymph nodes in 20-40% of cases and spleen, liver, bone, central nervous system (CNS), or kidney involvement in 2-8% of cases [1,6]. Hypercalcemia occurs in approximately 2-63% of these cases and clinically significant hypercalcemia occurs rarely [7]. As such, sarcoidosis is often skipped as a diagnosis when hypercalcemia is part of the initial presentation. Furthermore, a presentation of severe hypercalcemia (>14 mg/dL) is not typical of sarcoidosis [8]. In our case report, the patient presented with an initial serum calcium level of 18.1 mg/dL which made the initial diagnosis of sarcoidosis challenging.

Serum calcium levels play an important role in cardiac conduction, coagulation, and other cellular processes. The renal, endocrine, intestinal, and skeletal systems use the regulation of vitamin D (1,25-dihydroxyvitamin D), calcitonin, and PTH levels as feedback mechanisms to regulate extracellular calcium concentrations [2]. In most cases, hypercalcemia is mild (serum calcium 10-12 mg/dL) and detected incidentally in an asymptomatic patient. However, moderate-severe hypercalcemia with a serum calcium concentration above 13 mg/dL is usually symptomatic [9].

The etiology of moderate-severe hypercalcemia is often multifactorial, however, over 90% of cases are due to primary hyperparathyroidism or malignancy [10]. Lesser known causes include familial hypocalciuric hypercalcemia, granulomatous disorders, vitamin D intoxication, hyperthyroidism, vitamin A intoxication, multiple myeloma, and milk-alkali syndrome [11]. Hypercalcemia in granulomatous disorders is produced through a PTH-independent conversion of 25-hydroxyvitamin D to 1,25-dihydroxyvitamin D by 1 alpha-hydroxylase expressed by macrophages. This leads to a subsequent increase in activated vitamin D causing increased calcium absorption from intestines and resorption from bones. Among granulomatous disorders, sarcoidosis accounts for 1% of cases of hypercalcemia [12].

Moderate to severe hypercalcemia can have multisystem manifestations including constipation, nausea/vomiting, dysrhythmias, dehydration, polyuria, nephrolithiasis, weakness, muscle pain, behavior changes, or anxiety. Severe hypercalcemia can present dramatically in the form of neurological impairment, acute kidney injury, and AV blockade [13]. None of these symptoms were present in our patient despite her profound hypercalcemia.

The initial testing for hypercalcemia includes correction for albumin and attaining ionized calcium. This is followed by testing for serum PTH (to determine PTH dependence or independence) and a 24-hour urine calcium level. If these results are normal, other causes of hypercalcemia (apart from primary hyperparathyroidism or familial hypocalciuric hypercalcemia) should be considered. Another common cause of hypercalcemia is malignancy. Paraneoplastic syndromes commonly associated with malignancies of the pulmonary and renal systems involve the release of parathyroid hormone-releasing peptide, which then may be tested [14]. If a strong suspicion for malignancy exists, this should be appropriately correlated with imaging and/or the clinical presentation. To rule out other etiologies, 25-hydroxyvitamin D, vitamin A, thyroid hormone levels, ACE enzyme levels, urine protein electrophoresis, and serum protein electrophoresis must be similarly evaluated. 1,25-dihydroxyvitamin D (active form) is compared to 25-hydroxyvitamin D to evaluate for the possibility of granulomatous diseases such as sarcoidosis and hypersensitivity pneumonitis. However, studies have shown that 1,25-dihydroxyvitamin D may be normal with low 25-hydroxyvitamin D in sarcoidosis [15]. ACE enzyme levels are also tested in hypercalcemia but they are non-specific and can be elevated in PTH-dependent cancers, granulomatous disorders, silicosis, Gaucher’s disease, diabetes, and many more [16]. Hence, the diagnosis of hypercalcemia in sarcoidosis is difficult as the presentation can mimic other granulomatous diseases and malignancies. Radiological imaging and attention to detail of clinical presentation may be used to guide the diagnosis. Sarcoidosis commonly presents with hilar lymphadenopathy (90-95% of cases), followed by perihilar micronodules, opacities, and other fibrotic changes [17]. However, these findings can often mimic other parenchymal lung disorders such as idiopathic interstitial pneumonia, hypersensitivity pneumonitis, vasculitis, and lipoid pneumonia [18]. The confirmation of sarcoidosis requires biopsy from specific sites, such as lymph nodes, kidneys, lungs, or skin, based on the site of involvement.

In this case, the absence of hilar lymphadenopathy on CT imaging with an abnormally high elevation in calcium made the initial diagnosis of sarcoidosis unlikely. Moreover, the presence of anemia, hypercalcemia, and acute kidney failure made multiple myeloma a strong potential differential. However, this would not have explained the patient’s symptoms of long-standing dyspnea or the non-specific ground glass opacities identified on pulmonary imaging. The presence of normal PTH levels despite marked hypercalcemia made primary hyperparathyroidism a possible contributing factor towards hypercalcemia, as severely elevated serum calcium levels should have caused PTH to be low or suppressed. Further imaging with a sestamibi scan showed questionable findings of a parathyroid adenoma. A thyroid ultrasound was recommended to determine any correlation with the location of the findings, however, it did not reveal any parathyroid abnormalities. Furthermore, 1,25-dihydroxyvitamin D levels were elevated while 25-hydroxyvitamin D and ACE levels were normal leading to the suspicion of conversion from granulomatous disease. Finally, a bronchoscopy was performed and a biopsy taken showed non-necrotizing granulomas and chronic inflammation with a CD4:CD8 ratio of 4.5:1, pointing towards sarcoidosis as the primary cause of hypercalcemia.

Severe hypercalcemia is initially managed with intravenous bisphosphonates, IV fluids, and calcitonin to induce rapid correction [19]. However, the treatment of hypercalcemia in sarcoidosis is with corticosteroids. Steroids not only act as anti-inflammatory in sarcoidosis but also inhibit the 1 alpha-hydroxylase activity of macrophages. They also decrease calcium absorption from the gut and inhibit bone resorption by osteoclasts. Hypercalcemia is an indication of steroid initiation for patients with sarcoidosis even in the presence of minimal symptoms. Cases of steroid-resistant hypercalcemia in sarcoidosis have been documented and may be substituted with ketoconazole and infliximab but further studies are required for conclusive evidence [20].

## Conclusions

This case emphasizes the importance of recognizing atypical presentations of sarcoidosis, particularly in cases of severe hypercalcemia. It highlights the limitations of relying solely on serological markers and imaging studies, advocating for a thorough and individualized diagnostic evaluation to prevent delays in identifying and treating underlying medical conditions. Clinicians should exercise caution when interpreting diagnostic algorithms, acknowledging their inherent limitations in certain clinical scenarios.
